# Hypofractionated Prostate Radiotherapy with or without Conventionally Fractionated Nodal Irradiation: Clinical Toxicity Observations and Retrospective Daily Dosimetry

**DOI:** 10.1155/2012/546794

**Published:** 2012-06-28

**Authors:** Andrew M. McDonald, Justin M. Bishop, Rojymon Jacob, Michael C. Dobelbower, Robert Y. Kim, Eddy S. Yang, Heather Smith, Xingen Wu, John B. Fiveash

**Affiliations:** ^1^Department of Radiation Oncology, Hazelrig-Salter Radiation Oncology Center, University of Alabama at Birmingham, 1700 6th Avenue South Birmingham, Birmingham, AL 35249, USA; ^2^Birmingham Southern College, 900 Arkadelphia Road, Birmingham, AL 35254, USA

## Abstract

*Purpose*. To evaluate toxicity associated with the addition of elective nodal irradiation (ENI) to a hypofractionated regimen for the treatment of prostate cancer. *Methods and Materials*. Fifty-seven patients received pelvic image-guided IMRT to 50.4 Gy in 28 fractions with a hypofractionated simultaneous boost to the prostate to 70 Gy. Thirty-one patients received prostate-only treatment to 70 Gy in 28 fractions. *Results*. Median followup was 41.1 months. Early grade ≥2 urinary toxicity rates were 49% (28 of 57) for patients receiving ENI and 58% (18 of 31) for those not (*P* = 0.61). Early grade ≥2 rectal toxicity rates were 40% (23 of 57) and 23% (7 of 31), respectively (*P* = 0.09). The addition of ENI resulted in a 21% actuarial rate of late grade ≥2 rectal toxicity at 4 years, compared to 0% for patients treated to the prostate only (*P* = 0.02). Retrospective daily dosimetry of patients experiencing late rectal toxicity revealed an average increase of 2.67% of the rectal volume receiving 70 Gy compared to the original plan. *Conclusions*. The addition of ENI resulted in an increased risk of late rectal toxicity. Grade ≥2 late rectal toxicity was associated with worse daily rectal dosimetry compared to the treatment plan.

## 1. Introduction

Hypofractionated treatment regimens for clinically localized prostate cancer remain a topic of debate as large prospective trials such as RTOG 0415 continue to mature. The large retrospective series from Cleveland Clinic as well as recently published phase III data by Arcangeli et al. indicate that hypofractionated RT is well-tolerated and offers high rates of biochemical control [1, 2]. However, most published series focus primarily on patients with a relatively low risk of lymph node involvement and have, therefore, restricted treatment to the prostate and seminal vesicles only.

Traditionally, patients with high-risk prostate cancer receive whole-pelvic radiotherapy (WPRT), and such regimens have formed the backbone of large-scale clinical trials such as RTOG 8531, RTOG 9202, and EORTC 22863 [3–6]. Though conventionally fractionated WPRT has been safely delivered on a number of clinical trials, little data exists concerning treatment of the pelvic lymph nodes as part of a hypofractionated regimen. To date, there has only been one phase III trial which utilized hypofractionated WPRT for patients with high-risk disease [7]. While the short-term toxicity results of this study are promising, a detailed report of the long-term toxicities is pending.

Advances in radiation delivery such as intensity-modulated radiotherapy (IMRT) and image-guided radiotherapy (IGRT) have resulted in improved dose distribution and avoidance of normal structures during the treatment of prostate cancer [8, 9]. McCammon et al. utilized IMRT to show the feasibility of treating the pelvic lymph nodes as part of a hypofractionated regimen utilizing a simultaneous integrated boost to the prostate [10]. This technique, with the addition of IGRT and nodal dose escalation, was further investigated by Adkison et al. as a phase I study [11].

Our aim in this work was to perform clinical toxicity analysis of pelvic image-guided IMRT with a hypofractionated simultaneous integrated prostate boost compared to hypofractionated prostate-only radiotherapy. We hypothesized that treatment of the pelvic lymph nodes will result in increased toxicity compared with hypofractionated treatment to the prostate only. In addition, we aimed to perform retrospective daily dosimetry for patients who develop Grade 2 or higher late toxicities to correlate dose-levels to rectum with toxicity.

## 2. Materials and Methods

### 2.1. Inclusion Criteria

The records of patients receiving hypofractionated external beam radiation for the treatment of clinically localized prostate cancer at UAB since 2004 were reviewed. All patients meeting the following requirements were included in the analysis: external beam radiation with a total dose of 70 Gy to the prostate delivered in fractions of 2.5 Gy by IMRT, daily CT-based image-guidance, no previous treatment other than hormonal ablation, and followup ≥1 year. The study was approved by the University of Alabama at Birmingham Institutional Review Board.

### 2.2. Treatment Planning and Delivery

CT simulation was performed in the supine position with a lower extremity form in place with patients instructed to have a full bladder and empty rectum. If significant stool was noted in the rectum, patients were simulated a second time after a bowel movement. The rectum was contoured as a solid organ from the level of the ischial tuberosities inferiorly, to the rectosigmoid junction superiorly. Other avoidance structures contoured included the entire bladder (with contents) and femoral heads and greater trochanters.

The prostate clinical target volume (CTV_1_) was defined as the prostate along with any visible areas of tumor extension. The planning target volume for the prostate (PTV_1_) consisted of the CTV_1_ as well as a 7 mm extension in all directions, except posteriorly where the extension was 4-5 mm. The seminal vesicle CTV (CTV_2_) was defined as the entirety of the seminal vesicles. The PTV_2_ consisted of the CTV_2_ plus a 7 mm extension in all directions, except posteriorly where the extension was 4 mm. The nodal target volume (CTV_3_) was generated by contouring a 7 mm extension around the internal iliac, external iliac, and common iliac vessels to the level of mid-S1 in order to approximate a beam aperture at the level of the L5-S1 junction. The PTV_3_ was then generated by extending the CTV_3_ 7 mm in the lateral directions and 9 mm anteriorly and posteriorly.

Ten patients with low-risk cancer, as defined by the NCCN Guidelines, were prescribed 70 Gy to the PTV_1_ alone, delivered in 28 daily fractions of 2.5 Gy [12]. Twenty-one patients with low- or intermediate-risk cancer were prescribed 56 Gy to the PTV_2_ in 28 daily fractions of 2.0 Gy with a simultaneous integrated boost to the PTV_2_ to 70 Gy. All high-risk patients, as well as 13 intermediate-risk patients (with a calculated risk of 10% or more for pelvic nodal spread by Partin nomograms [13]), were prescribed 50.4 Gy to the PTV_3_ with simultaneous 56 Gy to the PTV_2_ and 70 Gy to the PTV_1_ all delivered simultaneously over 28 fractions of 1.8 Gy, 2.0 Gy, and 2.5 Gy, respectively.

Rectal constraints limited the volume receiving ≥60 Gy to the lesser of 10 cc or 10% of the total rectal volume. There was no maximum point dose for the rectum. No predefined bladder constraints were utilized, but high doses were minimized on an individualized basis. IMRT plans were then generated using inverse planning by TomoTherapy TomoAdaptive software or Varian Eclipse software. TomoAdaptive plans were required to deliver the whole of the prescription dose to a minimum of 95% of the PTV. Eclipse plans were required to deliver 95% of the prescription dose to the whole of the PTV. The PTV dose requirements were compromised if rectal constraints could not be met.

Treatment was delivered by a TomoTherapy machine or Varian 2100 linear accelerator. Daily image guidance was performed by megavoltage CT (TomoTherapy) or cone-beam kilovoltage CT (Varian) prior to each fraction. For CT-based image guidance, the alignment was performed to the prostate-rectal interface via a rigid translation of the plan. Hormonal ablation therapy typically began 3 months prior to the start of RT.

### 2.3. Followup

Patients were seen for return visits every 4 months for the first two years following RT, and every 6 months thereafter. Patients were assessed for urinary and rectal toxicity at each visit using the Common Terminology Criteria for Adverse Events 4.0 [14]. Only the highest grade early and late toxicities for each patient were taken into account for the analysis. Late toxicity was defined as new symptoms appearing greater than 3 months from the completion of RT. Early toxicities continuing beyond 3 months from the completion of RT were only recorded as early toxicity. In general, patients with rectal bleeding were initially treated with steroid suppositories, and those refractory to therapy were referred for endoscopic laser procedure. Patients with diarrhea more than twice per week were prescribed antidiarrheal medications.

### 2.4. Retrospective Daily Dosimetry

We aimed to perform retrospective daily dosimetry of the rectum for patients who recorded grade ≥2 late rectal toxicity. Utilizing a TomoTherapy treatment planning workstation and Planned Adaptive software, the rectum was contoured on each daily megavoltage CT scan and reviewed for agreement by two investigators. The actual dose delivered to the rectum was calculated for each fraction. For each patient, the cumulative dose-volume histogram (DVH) was calculated by summing the daily DVHs. The cumulative delivered rectal DVHs were compared with cumulative calculated rectal DVHs.

### 2.5. Statistical Methods

For the analysis, patients were stratified into groups receiving prostate-only radiotherapy (PORT) or WPRT based on whether or not ENI was delivered. The PORT group was not subdivided further based on whether or not patients received treatment to the seminal vesicles. Statistical analysis of the data was performed using IBM SPSS Statistics 15.5 software. The actuarial rates of progression-free and toxicity-free survival were calculated using the Kaplan-Meier method. Log-rank testing was used to determine statistically significant differences between groups. Toxicity-free survival was calculated from the beginning of RT.

## 3. Results

### 3.1. Pretreatment Characteristics

 Eighty-eight patients met the inclusion criteria for this analysis. Thirty-one patients received treatment limited to the prostate and seminal vesicles, and 57 received additional treatment to the pelvic lymph nodes. Seventy-one percent of patients received either adjuvant or neoadjuvant hormonal ablation. A summarized list of pretreatment characteristics by treatment regimen is presented in Table 1. Eighty-eight percent (50 of 57) of patients receiving ENI also received hormonal ablation therapy, compared to 39% (12 of 31) of those not receiving ENI (*P* < 0.01).

### 3.2. Acute Toxicity

The frequency of acute toxicity events divided by treatment type is presented in Table 2. Of the patients receiving PORT, 23% (7 of 31) experienced acute grade ≥2 GI toxicity and 58% (18 of 31) experienced acute grade ≥2 GU toxicity. WPRT treatment resulted in 40% (23 of 57) of patients experiencing early GI toxicity and 49% (28 of 57) of patients experiencing early GU toxicity. The difference in acute toxicity associated with the addition of ENI was not statistically significant. 

### 3.3. Late Toxicity

**Figure 1:**
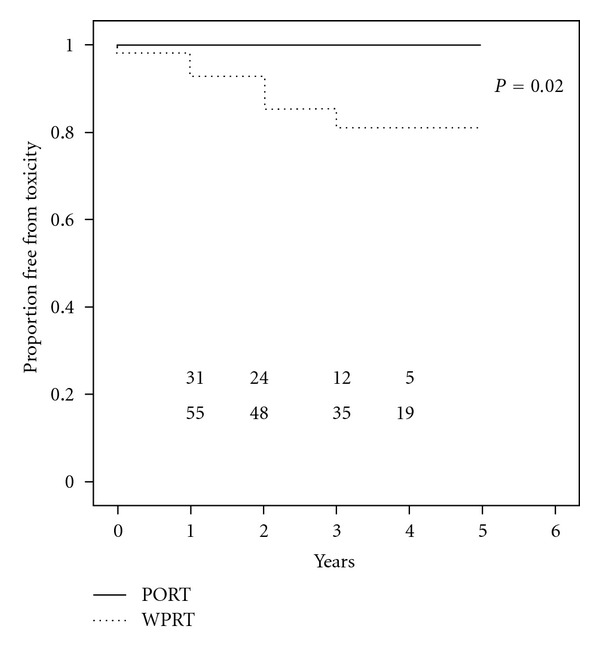
Kaplan-Meier plot of freedom from late rectal grade ≥2 toxicity.

The median followup was 41.1 months, ranging from 12 to 74 months. The rate of late grade ≥2 rectal toxicity was 18% (10 of 57) in the WPRT group and 0% in the PORT group. There was only one grade 3 late toxicity event and there were no grade 4 toxicity events. A Kaplan-Meier plot of freedom from late rectal toxicity is presented in Figure 1. At 4 years, the actuarial rate of grade ≥2 rectal events was 21% for the WPRT group and 0% for patients receiving PORT. This difference was statistically significant by log-rank testing (*P* = 0.02).

The rate of late grade 3 urinary toxicity was 5% (3 of 57) in the WPRT group and 0% in the PORT group. Two of these events were urethral strictures requiring mechanical dilatation and the third consisted of a single episode of gross hematuria. There were no grade 4 events. At 4 years the actuarial rate of grade 3 urinary events was 7% for the WPRT group and 0% for patients receiving PORT (*P* = 0.29).

### 3.4. Retrospective Daily Dosimetry

**Figure 2:**
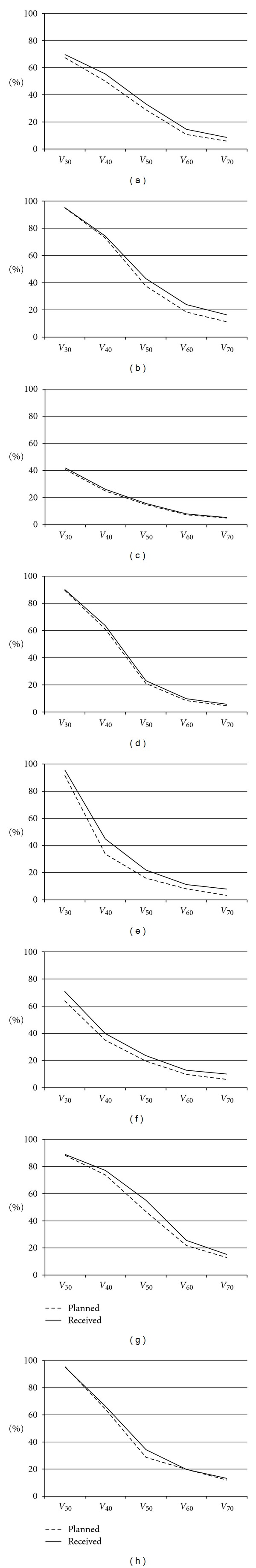
Average actual versus planned rectal DVHs for 8 patients experiencing late grade 2 or greater rectal toxicity.

Daily dose reconstruction was performed for 8 of the 10 patients who developed late grade ≥2 rectal toxicity. Daily CT scans of the remaining 2 patients were unavailable due to data corruption. A total of 224 scans were contoured. The combined average daily deviation of the actual rectal volume from the planned volume was 12.7% (individual patient averages ranged from 3.5% to 29.6%).  Figure 2 illustrates the actual versus planned rectal DVHs of these 8 patients. The actual *V*_30_, *V*_40_, *V*_50_, *V*_60_, and *V*_70_ were higher compared to the planned volumes for each patient. In particular, the cumulative *V*_70_ was 2.67% more than the planned volume. Eighty-eight percent of the individual fractions delivered a higher *V*_70_ than originally planned. A detailed comparison between planned and actual volumes is presented in Table 3.

## 4. Discussion

 The role of ENI in the context of high-dose prostate RT and androgen deprivation is controversial. However, whole pelvic irradiation has traditionally been used to treat men with high-risk prostate cancer, and is still widely practiced. Though hypofractionated prostate irradiation is gaining popularity, the use of ENI as part of a hypofractionated treatment regimen remains a relatively unexplored area. This retrospective toxicity analysis constitutes the first series comparing the addition of ENI between two groups of patients receiving hypofractionated radiotherapy.

The toxicity of hypofractionated PORT has been described previously, in retrospective series and prospective studies [1, 2, 15]. Investigators at the Cleveland Clinic reported outcomes among patients treated using 70 Gy in 28 fractions to the prostate (with the majority receiving treatment to the seminal vesicles as well). Sixty percent of patients also received additional androgen deprivation. This treatment resulted in a 9% rate of early grade 2 GI toxicity and an 18% rate of early GU toxicity. The actuarial rate of late grade ≥2 rectal complications was 6.1% at 5 years [1]. The hypofractionated arm of the phase III trial by Arcangeli et al. consisted of patients receiving 62 Gy in 20 fractions; all patients received neoadjuvant hormonal ablation. This study reported 35% acute grade 2 GI and 47% acute grade ≥2 GU toxicities. The actuarial rate of late grade ≥2 rectal toxicity was 17% at 3 years [2, 15]. In comparison, we observed acute GI and GU grade 2 toxicity rates of 23% and 58%, respectively, and an actuarial rate of grade ≥2 rectal toxicity of 0% at 4 years. The variations in toxicity results among these studies may be explained by their differing fractionation schedules and toxicity scales used. Large-scale multi-institutional toxicity data among patients receiving hypofractionated RT to the prostate will be available as RTOG 0415 nears maturity.

 In contrast to PORT, few studies have examined the toxicity of pelvic treatment with a hypofractionated simultaneous integrated boost to the prostate. McCammon et al. performed a retrospective analysis of 30 patients receiving 70 Gy to the prostate in 28 fractions with a simultaneous prescription of 50.4 Gy to the pelvic lymph nodes at 1.8 Gy per fraction. Thirty-seven percent and 64% of these patients developed early GI and GU toxicity. The actuarial rate of late grade ≥2 GI events was 13.4% at 6 years, with one patient experiencing a grade 4 event [10]. In a phase I study, Adkison et al. delivered 70 Gy to the prostate and 56 Gy (2.0 Gy per fraction) to the pelvic lymph nodes over 28 fractions. The rates of clinically significant early GI and GU toxicity were 32% and 37% respectively. An 8% crude rate of late rectal toxicity was reported [11]. A slightly different fractionation scheme was utilized by Quon who prescribed 45 Gy to the pelvic basin in 25 fractions with a simultaneous boost to the prostate to a total of 67.5 Gy. This treatment resulted in early rectal and urinary toxicity rates of 37% and 39%. The crude rate of late rectal events was 7% [16].

 A phase III randomized trial by Pollack et al. was begun in 2002 comparing hypofractionated and conventionally fractionated IMRT for patients with intermediate to high-risk prostate cancer. The prostate was treated to either 70.2 Gy in 26 fractions or 76 Gy in 38 fractions. The investigators also prescribed nodal irradiation to patients with high-risk cancer, delivered simultaneously to a total dose of 50 or 52 Gy in the hypofractionated group and 56 Gy in the conventionally fractionated group. Early published results of the first 100 men enrolled showed no difference in acute toxicity between the two treatment regimens [7]. The 5-year cumulative biochemical failure rate was 15.3% for the hypofractionated group and 15.4% for the conventionally fractionated group. The rate of grade ≥2 late GI and GU toxicities was 4.1% and 8.9% for the conventionally fractionated group and 5.9% and 13.8% for the hypofractionated group [17]. A detailed publication of these results is forthcoming.

 Conventionally fractionated pelvic radiation has also been extensively studied in prospective manner. The major large scale multi-institutional trials, such as RTOG 92-02, RTOG 94-13, and the phase 3 trial by Warde et al. utilized prostate doses up to 70 Gy at most [4, 6, 18]. Assuming an *α*/*β* of 3.0 for rectal tissue, the biologically equivalent dose (BED) of the hypofractionated treatment to the prostate in this study is 77 Gy in terms of 2.0 Gy equivalents, or 80.2 Gy in terms of 1.8 Gy equivalents. Additionally, each of the aforementioned studies utilized a conventional four-field box technique whereas patients in our study received pelvic radiation by image-guided IMRT. These differences make toxicity comparisons between our study and the aforementioned studies difficult. However, there have been retrospective series published utilizing pelvic IMRT with a dose-escalated prostate boost. Deville et al. performed a retrospective analysis of 30 patients receiving pelvic IMRT to a dose of 45 Gy in 1.8 Gy fractions followed by a prostate boost to a total dose of 79.2 Gy [19]. This treatment resulted in a 50% (15 of 30) rate of early grade 2 GI toxicity and a 20% (6 of 30) rate of grade ≥2 late GI toxicity. The Fox Chase phase III trial by Pollack et al. utilized conventionally fractionated pelvic IMRT for patients with a higher risk of lymph node involvement based on the Roach formula who were randomized to the conventionally fractionated arm [7]. However, the long-term toxicity results from this group are pending.

The addition of ENI to our hypofractionated regimen did not increase the rate of early toxicity, but was associated with a statistically significant (*P* = 0.02) increase in late rectal toxicity, with an actuarial rate of 21% at 4 years. However, it is notable that there was only one grade 3 event. By performing retrospective daily dosimetry and calculating daily rectal DVH curves, we were able to confirm that the patients who developed late GI toxicity received a high dose of radiation to larger volumes of the rectum compared to the original treatment plan. This likely occurred due to the inherent difficulty associated with accounting for the inter- and intrafractional motion of the prostate including organ deformation with rectal filling. We calculated a mean change in rectal volume of 12.7% between fractions. Though the toxicity rates in this study are low and comparable to other studies, our dosimetric studies confirm that with increased attention to limiting dose to the rectum, it may be possible to reduce late rectal and bladder toxicities further.

 Limitations of our study include a relatively small number of patients and the retrospective nature of the analysis. The treatment methods within the PORT group were slightly variable with some patients receiving treatment to the seminal vesicles as well as the prostate. A much larger proportion of patients receiving pelvic RT also received neoadjuvant hormonal ablation compared to the PORT group, possibly contributing to the difference in late rectal complication rates. The results of RTOG 94-13 showed conventionally fractionated WPRT combined with neoadjuvant hormonal ablation to be associated with an increased frequency of grade ≥3 late rectal complications compared to the other 3 arms in the study [6]. The retrospective daily dosimetry of patients experiencing late rectal toxicity suggests that interfractionational changes in rectal position and volume contributed to these events. However, retrospective daily dosimetry for patients who did not experience late complications is required to confirm this hypothesis, and this data is currently being acquired. The median followup of 41.1 months is relatively short; however, as pointed out in other publications, the rate of rectal toxicity tends to plateau 2 years from the completion of RT [1, 15].

## 5. Conclusion

Pelvic image-guided IMRT with a hypofractionated simultaneous boost to the prostate did not result in increased rates of acute toxicity compared to hypofractionated treatment of the prostate alone. However, ENI was associated with a statistically significant increase in the probability of late rectal toxicity. Grade ≥2 late rectal toxicity was associated with worse daily rectal dosimetry compared to the treatment plan. However, even with ENI, the rate of late rectal toxicity remains moderate and may be acceptable given a high risk of pelvic lymph node involvement. Future prospective work is required to confirm these findings.

## Figures and Tables

**Table 1:** Pretreatment characteristics.

		PORT (*n* = 31)	WPRT (*n* = 57)	*P*
Age (years)		71.9	73.1	0.57

PSA	Mean	6.9	23.9	
	<10 ng/mL	24	20	0.015
	10–20 ng/mL	7	17	
	≥20 ng/mL	0	21	

Gleason score	Mean	6.42	7.72	
	≤6	18	6	<0.01
	=7	13	21	
	≥8	0	31	

NCCN risk group	Low	15	0	
	Intermediate	16	13	
	High	0	45	

Percent receiving hormonal ablation		39%	88%	<0.01

**Table 2:** Acute toxicity results.

	PORT	WPRT	*P*
Early grade ≥2 GI toxicity	7/31 (23%)	23/57 (40%)	0.09
Early grade ≥2 GU toxicity	18/31 (58%)	28/57 (49%)	0.61

**Table 3:** Detailed volumetric results of daily retrospective dosimetry calculations.

	*V*_70_	*V*_60_	*V*_50_	*V*_40_
Planned rectal dose (all patients)	7.59% ± 3.78%	13.03% ± 5.90%	26.69% ± 11.13%	51.92% ± 18.91%
Delivered rectal dose	10.28% ± 4.18%	15.70% ± 6.58%	31.27% ± 12.97%	55.95% ± 17.84%
Average difference	+2.69%	+2.67%	+4.58%	+4.03%
